# Enhanced Microplastic Flotation: Unraveling the Role of Bubble‐Chain Hydrodynamics via PIV Analysis

**DOI:** 10.1002/wer.70374

**Published:** 2026-04-08

**Authors:** Hyeok Jun Nam, Sung Jun Han, Jeong Jae Kim

**Affiliations:** ^1^ Department of Mechanical Engineering Hanbat National University Daejeon Korea; ^2^ Division of Environmental Science and Engineering Pohang University of Science and Technology Pohang Korea

**Keywords:** bubble dynamics, bubble–particle interaction, dissolved air flotation, microplastics, particle image velocimetry

## Abstract

Understanding the transport behavior of microplastic particles in flotation systems is critical for enhancing their removal efficiency in wastewater treatment processes. Here, we investigated the particle‐lifting effect of a bubble‐chain system on polystyrene (PS) particles under continuous rising flow conditions. A custom‐built chamber equipped with syringe‐driven needle injectors generated successive air bubbles with mean equivalent diameters of 3.21 and 2.81 mm. As the number of bubbles (*N*) increased from 1 to 10, the nondimensionalized vertical displacement (*L*_*z*_) of PS particles increased from 5.29 to 7.66 and 4.53 to 6.07 for 3.21 and 2.81‐mm bubbles, respectively. Correspondingly, the nondimensionalized total displacement (*L*_*total*_) increased from 5.58 to 8.31 and 4.78 to 6.52, showing maximum increases of 44.8% and 48.7%, respectively. As the number of bubbles increased, the axial motion of the particles was significantly enhanced, which was attributed to the vertical expansion of the flow field, the increase in vorticity, and the formation of asymmetric flow structures as identified through particle image velocimetry (PIV) analysis. The formation of overlapping vortices within bubble‐chain systems substantially broadens the effective hydrodynamic field, facilitating improved flotation of microplastic particles. This extended flow structure provides a quantifiable framework for enhancing the efficiency of dissolved air flotation (DAF) technologies, particularly in applications targeting microplastic separation.

## Summary

- Bubble‐chain dynamics markedly enhanced the axial transport of microplastic particles.
- Superimposed vortices broadened flow fields, thereby increasing flotation efficiency.
- Hydrodynamics insights from bubble chains establish a basis for optimizing DAF systems.

## Graphical abstract

A rising bubble‐chain generates overlapping vortex structures that enhance the upward transport and flotation of microplastic particles. This study identifies bubble‐chain hydrodynamics as a key mechanism for improving microplastic removal efficiency in dissolved air flotation processes.

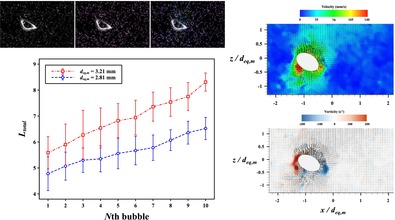

## 1 Introduction

Since the 1950s, plastics waste has been increasingly recognized as a major global pollutant, primarily due to the rapid escalation of plastic production, the absence of adequate disposal regulations, and the intensification of human activities (Horton and Barnes 2020; Ritchie 2022). Microplastics (MPs), typically defined as plastic debris smaller than 1 mm, are broadly categorized into two classes according to their origin (Browne et al. 2011; Claessens et al. 2013; Hartmann et al. 2019). Primary MPs are intentionally manufactured and directly introduced into the environment via industrial raw materials, resin pellets, and personal care products (Cole et al. 2011; Zhang et al. 2018). In contrast, secondary MPs are generated through the fragmentation of larger plastics because of mechanical abrasion, chemical weathering, and biological activity (Ryan et al. 2009; Thompson et al. 2004). Owing to their high dispersibility and mobility, MPs have been widely detected across diverse biological and environmental matrices (Hale et al. 2020; Horton et al. 2017), including sediments (Vaughan et al. 2017), marine ecosystems (Woodall et al. 2014), freshwater systems (Wang et al. 2017), polar regions (La Daana et al. 2018; Peeken et al. 2018), and even within human lung tissues (Jenner et al. 2022).

The ecological risks posed by MPs to marine organisms have been consistently highlighted in prior studies. Ingestion by aquatic fauna can lead to suffocation of false satiation, thereby suppressing normal feeding behavior and ultimately causing malnutrition and starvation (Cole et al. 2011). Furthermore, MPs may act as vectors for hazardous chemicals, raising concerns regarding the introduction of secondary toxicity into marine ecosystems (Wardrop et al. 2016). Experimental evidence further suggests that MPs are capable of trophic transfer within marine food webs, implying that their biological effects can propagate across multiple trophic levels (Farrell and Nelson 2013; Setälä et al. 2014). Such transfer has the potential to disrupt the structural integrity of both flora and fauna populations (Ivleva et al. 2017; Santana et al. 2017).

In response to the escalating problem, multiple physicochemical treatment technologies have been integrated into wastewater treatment processes, among which rapid sand filtration (RSF), granular activated carbon filtration (GACF), membrane bioreactors (MBRs), and dissolved air flotation (DAF) are considered representative. RSF has been widely employed due to its simple configuration and operational convenience, and it has achieved removal efficiencies of up to 98% for MPs smaller than 10 μm under drinking water treatment conditions (Chabi et al. 2024). However, when applied at full‐scale wastewater treatment facilities, its performance did not significantly differ from that of MBRs (Bayo et al. 2020). GACF, an adsorption‐based process, has demonstrated a maximum adsorption capacity of 6.33 mg/g for polystyrene (PS) nanoplastics (Arenas et al. 2021), and in continuous column systems treating highly concentrated synthetic wastewater, it has achieved polyethylene (PE, 40–48 μm) removal rates of up to 95.5% (Amirah Mohd Napi et al. 2023). Nonetheless, adsorption efficiency decreases markedly with increasing pollutant concentrations.

DAF, in contrast, operates on bubble–particle attachment and flotation principle, which is particularly advantageous for removing low‐density or nonspherical MPs due to their greater affinity for bubble attachment (Swart et al. 2022). Yet, this process is highly sensitive to operational factors such as the type and presence of coagulants, as well as bubble size and concentration. The introduction of coagulants consistently enhances the removal efficiency of large and low‐density MPs (Wang et al. 2021; Zhang et al. 2021). Moreover, compared to filtration‐based systems, DAF exhibits lower cumulative pressure drops, reduced backwashing demands, and higher operational stability under high‐turbidity and fluctuating flow conditions, making it a viable alternative or supplementary stage to sedimentation (De Somer et al. 2024). These process‐specific advantages have prompted numerous studies to investigate bubble–particle interaction mechanisms in DAF systems (Albijanic et al. 2014; Xing et al. 2017).

Despite the efforts, previous investigations—mostly conducted under controlled laboratory conditions—have provided only partial insights into bubble–particle interactions and have not fully captured the complex hydrodynamic environments generated by continuously rising bubble chain in real DAF operations. Although such flow structures are expected to influence MP behavior and flotation efficiency, systematic studies remain limited. Therefore, the present work experimentally reconstructs a continuously rising bubble‐chain system and examines its influence on MP dynamics using particle image velocimetry (PIV). The study aims to quantitatively evaluate how the flow field governs particle behavior and flotation performance. However, this research does not attempt process optimization; rather, it provides a fundamental dataset to support future design and operational improvements of DAF systems.

## 2 Experimental Apparatus and Methods

### 2.1 Characteristics of MPs

The MPs used in this study consisted of white polystyrene (PS) particles with a density of 0.9 g/cm^3^, and their actual morphology was visualized by scanning electron microscopy (SEM), as shown in Figure S1. PS was selected as the target particle because it is the third most frequently detected synthetic polymer in both marine and freshwater environments, following polyethylene (PE) and polypropylene (PP) (Erni‐Cassola et al. 2019; Li et al. 2018).

PE and PP possess relatively low densities, causing them to remain afloat on the water surface; this characteristic makes it difficult to maintain a uniform suspension or to precisely control hydrodynamic conditions under laboratory settings (Hidalgo‐Ruz et al. 2012; Kooi and Koelmans 2019). In contrast, PS exhibits stable suspension in water, providing more favorable physical properties for experiments related to flotation‐based separation processes (Hidalgo‐Ruz et al. 2012; Schwarz et al. 2019).

The particle size distribution of the MPs was measured using a laser diffraction particle size analyzer (Mastersizer 3000, Malvern Panalytical, UK) installed at Hanbat National University. The results are summarized in Table S1, while the corresponding graph showing a relatively broad size distribution is presented in Figure S2. The volume mean diameter, D[4,3], was determined to be 19.0 μm, which is a commonly used quantitative index that reflects the volumetric contribution of larger particles in particle size characterization.

### 2.2 Bubble‐Chain Generation and PIV Measurement System

**FIGURE 1:**
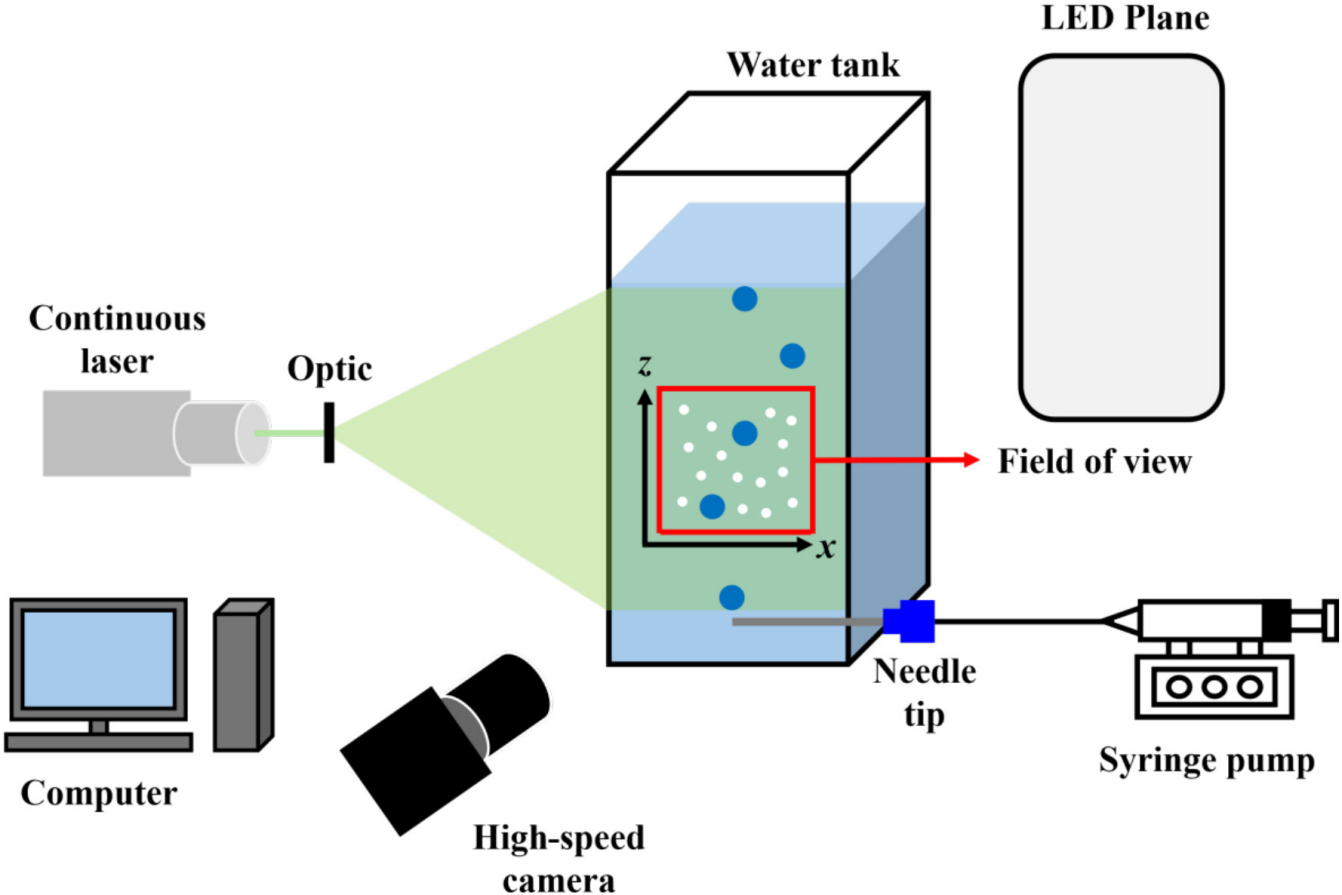
Schematic diagram of the experimental setup: shadowgraph imaging system used to visualize the shape and trajectory of rising bubbles, and setup used to measure the flow field around the bubble‐chain in the vertical (*x*–*z*) plane using particle image velocimetry.

A schematic of the experimental setup for bubble‐chain generation and the laser sheet illumination system used for PIV measurements is presented in Figure 1. The experiments were conducted in an open rectangular water tank with dimensions of 120 × 120 × 300 mm^3^, which was constructed with transparent acrylic panels on all four sides to ensure optical accessibility for PIV (Choi and Park 2021; Lee and Park 2017, 2022). After filling the tank with 3 L of distilled water (DAI HAN PHARM. CO., LTD, Korea), an MP suspension was prepared by dispersing 0.027 g of PS particles, corresponding to a volumetric fraction of *Φ* (=10^−5^). During the experiment, the water temperature was maintained at 25.9°C.

For bubble generation, two sterile hypodermic needles (Koreavaccine, Korea) with inner diameters of 0.32 and 0.23 mm, respectively, were employed. Both needles had an identical length of 60 mm and were fixed at a height of 5 mm above the tank bottom. They were connected to a syringe pump (Syringe ONE, New Era Instruments, USA) operating at a flow rate of 10 mL/min to continuously generate bubbles. A high‐speed camera (MINI UX, Photron, USA) was positioned 150 mm above the needle tips and recorded at 2000 fps with a resolution of 1280 × 720 pixels, yielding a calibrated spatial resolution of 27.2 μm/pixel.

On the opposite side of the camera, a white LED plane was installed to generate bubble shadows, while a continuous 2 W, 532‐nm green laser sheet was employed to illuminate PS particles for flow tracking. The acquired images were processed using the PIVview software (PIVview, PIVTEC, Germany). To extract instantaneous liquid‐phase velocity vectors, a conventional cross‐correlation algorithm was applied with an interrogation multigrid window of 16 × 16 pixels and an overlap of 50%.

In this study, PS particles were directly employed as tracer particles for PIV analysis, and no additional seeding particles were introduced. The suitability of this approach was assessed using the Stokes number (*St*), defined as the ratio of particle relaxation time to characteristic fluid time scale. Under the experimental conditions, the maximum *St* was approximately 1.01 × 10^−3^, which is well below the commonly accepted threshold (*St* < 0.1), indicating that the PS particles closely follow the liquid‐phase motion (Brandon and Aggarwal 2001). All experimental results were reported as the average values obtained from three replicate experiments.

### 2.3 Bubble Measurement

The equivalent diameter (*d*_*eq*_) of the bubbles generated from each needle was calculated using the relation: *d*_*eq*_ = (*d*_*long*_^*2*^ *d*_*short*_)^1/3^. Here, *d*_*long*_ and *d*_*short*_ represent the major and minor axes of the bubble, respectively. The aspect ratio (*χ*) was determined by *χ* = *d*_*long*_/*d*_*short*_. Liu et al. (2005) reported that bubbles rising in low‐viscosity fluids undergo continuous shape deformation and exhibit highly unstable trajectories. Similar behavior was observed in the current study, where the bubbles continuously changed shape during ascent, resulting in time‐dependent variations in both the *d*_*eq*_ and *χ*.

To ensure consistent analysis, bubbles were measured at the instant they passed the vertical center of the field of view (FOV) along the *z*‐axis, ensuring consistency in shape analysis. Further background on this measurement criterion is provided in Section 3.1. The calculation results are summarized in Tables S2 and S3. The mean equivalent diameter of bubbles (*d*_*eq,m*_) generated using the 0.32‐mm needle was determined to be *d*_*eq,m*_ = 3.21 mm, whereas for the 0.23‐mm needle, the corresponding value was *d*_*eq,m*_ = 2.81 mm, respectively. Bubble rising velocity (*U*_*b*_) is calculated as

(1) $$U_{b}=\frac{\sum_{i=1}^{n}\sqrt{{\left(x_{i}-x_{i-1}\right)}^{2}+{\left(z_{i}-z_{i-1}\right)}^{2}}}{\mathrm{\Delta t}}$$

where, *x*_*i*_ and *z*_*i*_ represent the horizontal (*x*‐direction) and vertical (*z*‐direction) positions of the bubble at frame *i*, respectively, and *n* is the total number of frames during the observed trajectory.

### 2.4 Image Analysis

To analyze the behavior of PS particles driven by bubbly flows, the open‐source image processing software Fiji was utilized (Schindelin et al. 2012). Particle detection was conducted using the Laplacian of Gaussian (LoG) algorithm, identifying regions with sharp intensity gradients by applying a second‐order derivative filter. This method is highly effective for detecting microscopic, blob‐like structures with near‐circular shapes (Tinevez et al. 2017).

**FIGURE 2:**
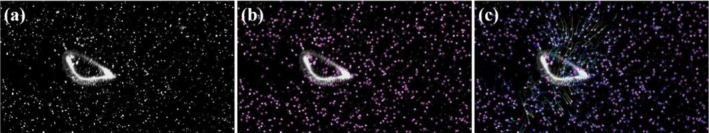
Procedure for detecting and tracking PS particles from high‐speed imaging. (a) Raw image obtained from the PIV experiment, (b) PS particles identified using the LoG detector, and (c) Particle trajectories reconstructed using the Simple LAP Tracker algorithm.

Subsequently, particle trajectories were reconstructed using a linear assignment problem (LAP)‐based algorithm allowing robust tracking of particle trajectories across frames, even when partial overlap or occlusion occurs (Ershov et al. 2022). Here, the LoG method was applied to raw high‐speed images (Figure 2a) to detect PS particles (Figure 2b), and the LAP algorithm was used to visualize their trajectories (Figure 2c). This analytical approach enabled the quantitative evaluation of the influence of the bubble‐chain system on PS particle transport.

### 2.5 Statistical Analysis

To quantify the effects of bubble number and bubble size on particle displacement, a multiple linear regression analysis was conducted. The analysis examined the contributions of the number of bubbles (*N*) and *d*_*eq*_ to particle displacement.

Particle displacement was defined as the dependent variable (*L*_*p*_), representing the horizontal displacement (*L*_*x*_), vertical displacement (*L*_*z*_), or total displacement (*L*_*total*_). The regression model is expressed as

(2) $$L_{p}=\beta_{0}+\beta_{1}N+\beta_{2}d_{\mathrm{eq}}$$

where *β*_*0*_ denotes the intercept, and *β*_*1*_ and *β*_*2*_ are the regression coefficients corresponding to *N* and *d*_*eq*_, respectively. The statistical significance of the regression model was evaluated using an *F*‐test, and the significance of individual regression coefficients was assessed using two‐tailed *t*‐tests. A significance level of *α* = 0.05 was applied throughout the analysis.

Model diagnostics were conducted to examine regression assumptions. The normality of residuals was tested using the Shapiro–Wilk test. Multicollinearity among the independent variables was assessed using the Variance inflation factor (VIF). All statistical analyses were performed using Python 3.12 with the statsmodels (v0.14.2) and scipy (v1.13.1) libraries.

## 3 Results

### 3.1 Bubble Dynamics

The motion of a rising bubble in a quiescent fluid is governed by a complex interplay among buoyancy, inertial, and viscous forces, and this behavior is generally characterized by the Reynolds number (*Re*) (Grace 1973). Here, *Re* is defined as

(3) Re=ρULμ

where, *ρ* is the density of water, *μ* is the dynamic viscosity of water, *U* is fluid velocity, and L is the characteristic length. To evaluate the *Re* of individual bubbles, *U* was taken as *U*_*b*_ and *L* was taken as *d*_*eq*_. Under the experimental conditions, the calculated *Re* ranged from 110–155. The corresponding *U*_*b*_ and the associated *Re* values are summarized in Tables 1 and 2. This range is sufficiently high to induce complex flow phenomena such as shape deformation and trajectory instability (Clift et al. 1978). Earlier studies widely reported that bubble path instability typically becomes pronounced only after the bubble ascended a certain distance (Duineveld 1995; Mougin and Magnaudet 2001; Wu and Gharib 2002).

**TABLE 1:** *U*_*b*_ and *Re* of individual bubbles constituting the bubble‐chain system when using the needle with an I.D. of 0.32 mm.

*N*th	1	2	3	4	5	6	7	8	9	10
*U*_*b*_ (mm/s)	39.4	37.9	36.7	37.7	39.1	37.1	40.2	40.2	39.8	39.3
*Re*	126	130	128	139	144	134	156	139	155	141

**TABLE 2:** *U*_*b*_ and *Re* of individual bubbles constituting the bubble‐chain system when using the needle with an I.D. of 0.23 mm.

*N*th	1	2	3	4	5	6	7	8	9	10
*U*_*b*_ (mm/s)	38.0	38.9	39.5	40.4	39.6	37.4	37.4	39.6	37.4	37.8
*Re*	115	110	116	118	122	120	130	129	123	130

**FIGURE 3:**
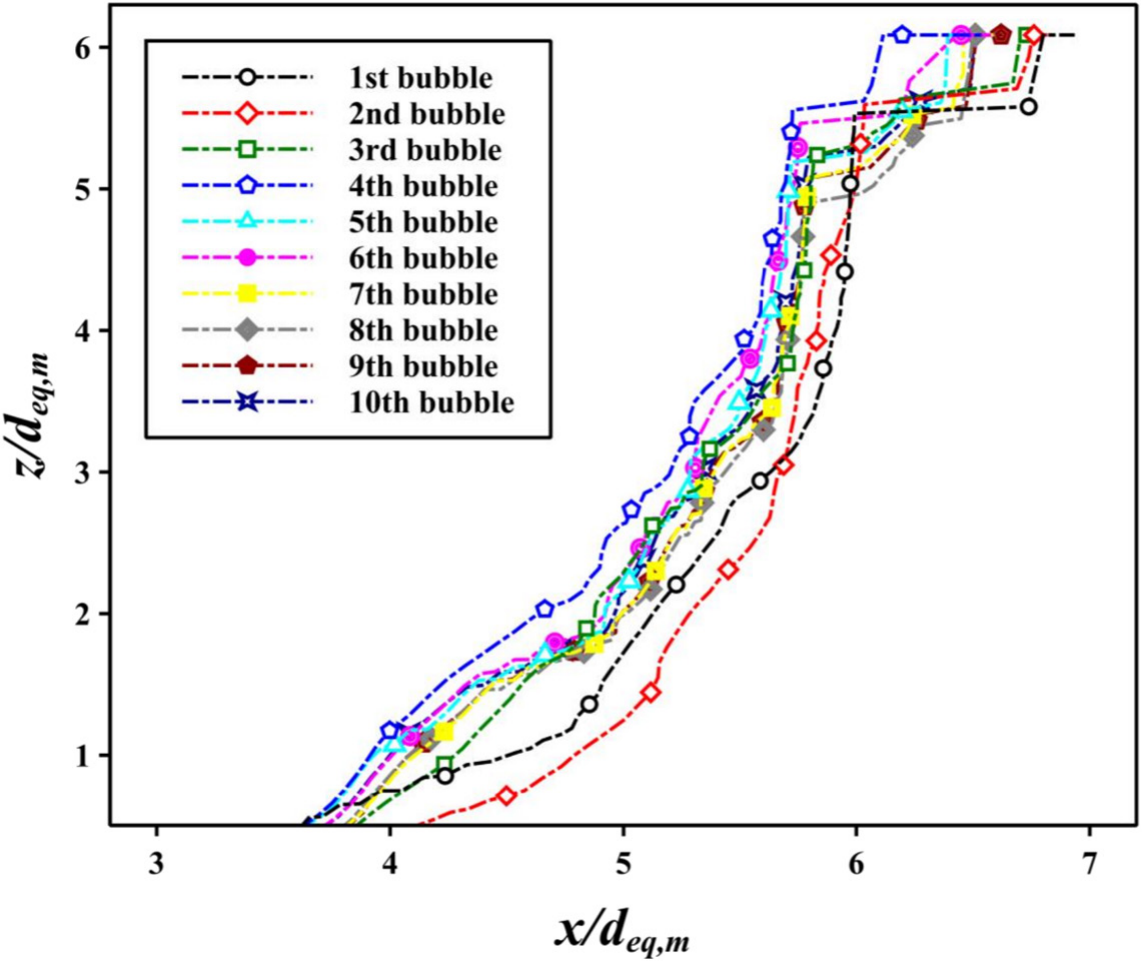
Rising trajectories of individual bubbles for the condition *d*_*eq,m*_ = 3.21 mm, obtained by tracking the centroid positions of each bubble.

All bubbles exhibited trajectory instability within the FOV, with this behavior consistently emerging beyond a specific axial location. As illustrated in Figure 3, every bubble involved in the experiment deviated from a linear vertical path due to lateral oscillations, indicating a nonlinear trajectory. Although there was slight variation in the initial onset of instability among individual bubbles, consistent path deviation was observed once the normalized vertical height exceeded *z* = 5.5 *d*_*eq,m*_.

### 3.2 Results of Axial Displacement of PS Particles

The key quantitative results for *L*_*x*_, *L*_*z*_, and *L*_*total*_ are summarized in Tables 3 and 4, which report representative values at *N* = 1 and *N* = 10 for both bubble sizes. To improve clarity and readability, the following description focuses on the overall trends and comparative behavior with increasing *N*, while detailed numerical values are provided in the tables and figures.

**TABLE 3:** Summary of *L*_*x*_, *L*_*z*_, and *L*_*total*_ values for PS particles in the bubble‐chain system with *d*_*eq,m*_ = 3.21 mm. Representative values at *N* = 1 and *N* = 10 are reported, along with the corresponding absolute and percentage increases relative to the single‐bubble condition. (Δ: value at *N* = 10—value at *N* = 1; %Δ: percentage increase relative to *N* = 1).

Parameter	*N* = 1	*N* = 10	Δ	% Δ
*L*_*x*_	1.78	3.22	1.44	80.9%
*L*_*z*_	5.29	7.66	2.37	44.8%
*L*_*total*_	5.59	8.31	2.72	48.7%

**TABLE 4:** Summary of *L*_*x*_, *L*_*z*_, and *L*_*total*_ values for PS particles in the bubble‐chain system with *d*_*eq,m*_ = 2.81 mm. Representative values at *N* = 1 and *N* = 10 are reported, along with the corresponding absolute and percentage increases relative to the single‐bubble condition.

Parameter	*N* = 1	*N* = 10	Δ	% Δ
*L*_*x*_	1.48	2.36	0.88	59.5%
*L*_*z*_	4.53	6.08	1.55	34.2%
*L*_*total*_	4.78	6.52	1.74	36.4%

*Note:* (Δ: value at *N* = 10—value at *N* = 1; %Δ: percentage increase relative to *N* = 1).

**FIGURE 4:**
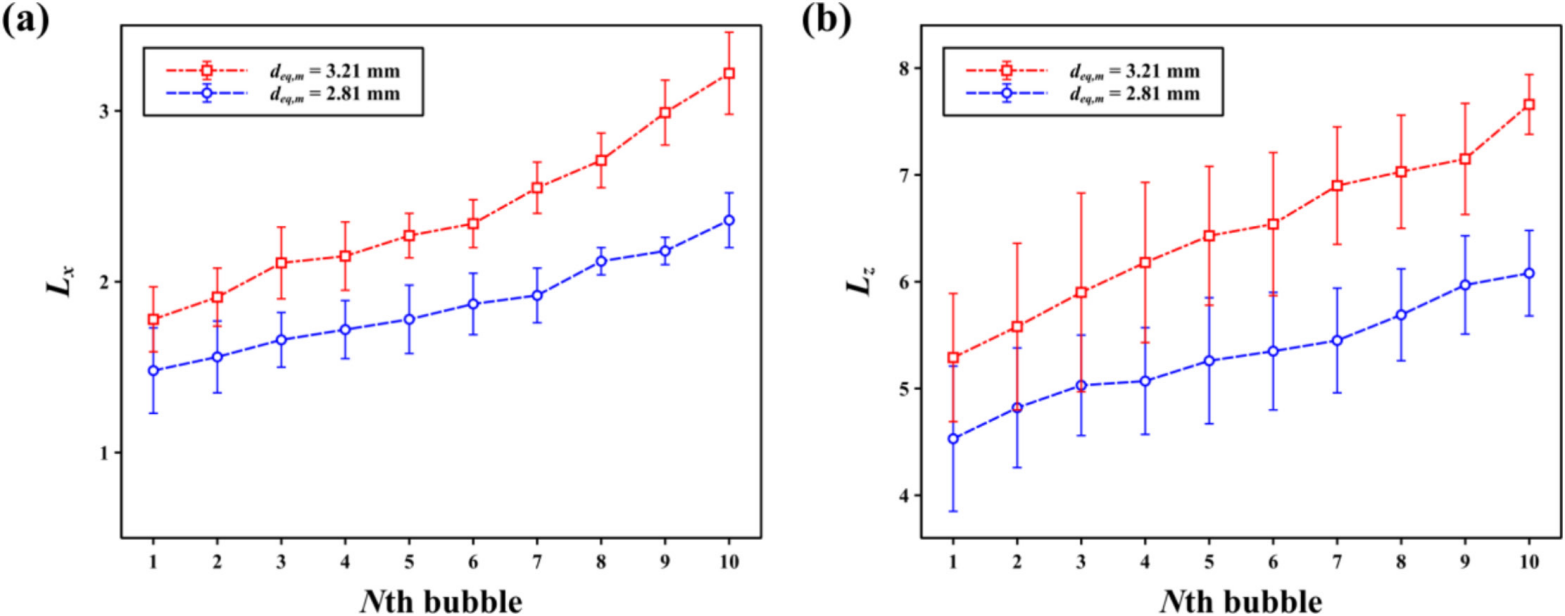
Nondimensionalized particle displacement induced by the bubble‐chain flow field as a function of bubble number *N*. (a) Horizontal displacement (*L*_*x*_) and (b) vertical displacement (*L*_*z*_) of a representative PS particle, normalized by the particle diameter, are shown for two mean equivalent bubble diameters, *d*_*eq,m*_ = 3.21 and 2.81 mm. Error bars indicate the standard deviation obtained from repeated measurements.

**FIGURE 5:**
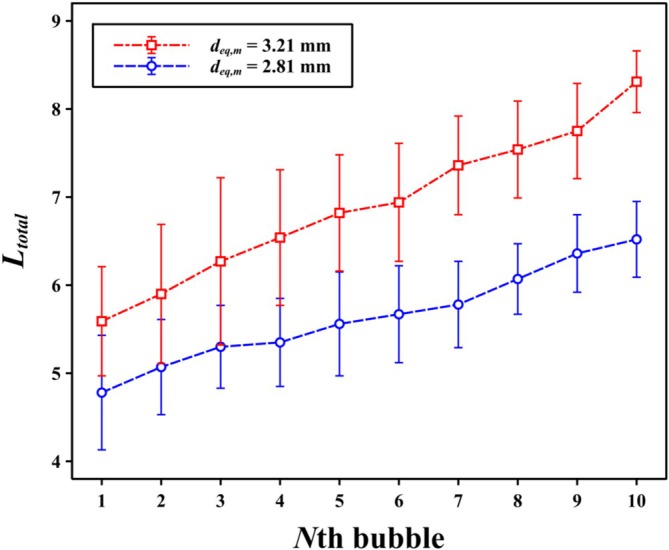
Nondimensionalized total displacement (*L*_*total*_) of a PS particle induced by the bubble‐chain system as a function of bubble number *N*. The total displacement is defined as the cumulative particle trajectory length combining both horizontal and vertical motions and normalized by the particle diameter. Data is presented for two mean equivalent bubble diameters, *d*_*eq,m*_ = 3.21 and 2.81 mm. Error bars represent the standard deviation obtained from repeated measurements.

*L*_*x*_ exhibited a gradual increase with N for both bubble sizes, followed by a more pronounced rise at higher *N* values (Figure 4a). This qualitative evolution was observed consistently for *d*_*eq,m*_ = 3.21 and 2.81 mm, indicating that the overall trend of *L*_*x*_ with increasing *N* is weakly dependent on bubble diameter. *L*_*z*_ increased monotonically with *N* for both cases (Figure 4b), although the rate of increase differed between the two bubble sizes, with larger bubbles showing a steeper growth. A similar increasing tendency was observed for *L*_*total*_ as a function of *N* (Figure 5).

### 3.3 Results of Statistical Analysis

Diagnostic tests indicated no major violations of regression assumptions. The Shapiro–Wilk test did not detect significant deviations from normality in the residuals for *L*_*x*_, *L*_*z*_, and *L*_*total*_ (*p* = 0.435, 0.060, and 0.163, respectively). Multicollinearity between the independent variables was low, with a VIF of 1.34 for both *N* and *d*_eq_, indicating no substantial collinearity within the linear modeling framework.

As summarized in Table 5, all regression models were statistically significant (*p* < 0.001). The *R* ^2^ were 0.698, 0.537, and 0.575 for *L*_*x*_, *L*_*z*_, and *L*_*total*_, respectively. These values indicate that *N* and *d*_*eq*_ jointly explained a considerable fraction of the variance in particle displacement, although a portion of variability remained unexplained.

**TABLE 5:** Results of multiple linear regression analysis for particle displacement.

Dependent variable	*R* ^2^	*F*‐test (*p*)	Independent Variable	Coefficient (*β*)	*t*‐test (*p*)
*L*_*x*_	0.698	< 0.001	Constant (*β*₀)	−0.989	
			*N* (*β*₁)	0.079	< 0.001
			d_eq_ (*β*_2_)	0.893	< 0.001
*L*_*z*_	0.537	< 0.001	Constant (*β*₀)	−0.668	
			*N* (*β*₁)	0.107	0.005
			d_eq_ (*β*_2_)	1.983	< 0.001
*L*_*total*_	0.575	< 0.001	Constant (*β*₀)	−0.946	
			*N* (*β*₁)	0.128	0.001
			d_eq_ (*β*_2_)	2.164	< 0.001

Both *N* and *d*_*eq*_ were statistically significant predictors across all displacement components. These results quantitatively support the experimental observation that particle displacement increases with bubble number and bubble size within the examined parameter range. However, the regression analysis reflects linear associations captured by the model and does not, by itself, establish mechanistic causality.

### 3.4 Characteristics of Flow Fields in Bubble‐Chain System

Rising bubbles generated localized flow fields in their vicinity, and the spatial extent of these flow structures increased progressively with bubble number. In this study, the flow field was analyzed at the instant when the bubble center reached the normalized position *z*/*d*_*eq,m*_ = 0 within the FOV.

**FIGURE 6:**
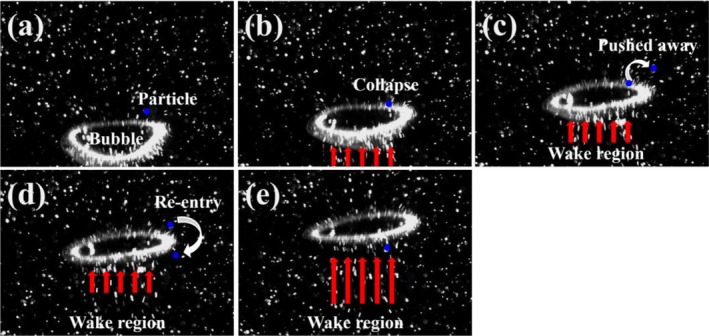
Time‐sequential high‐speed images illustrating the qualitative bubble–particle interaction mechanism induced by collision during the ascent of a single bubble. Frames (a) through (e) are arranged in chronological order with a constant time interval of *Δt* = 2.5 ms between consecutive frames. This figure was prepared with reference to the mechanism reported by Choi and Park (2022) and does not reproduce or reuse their data or results.

**FIGURE 7:**
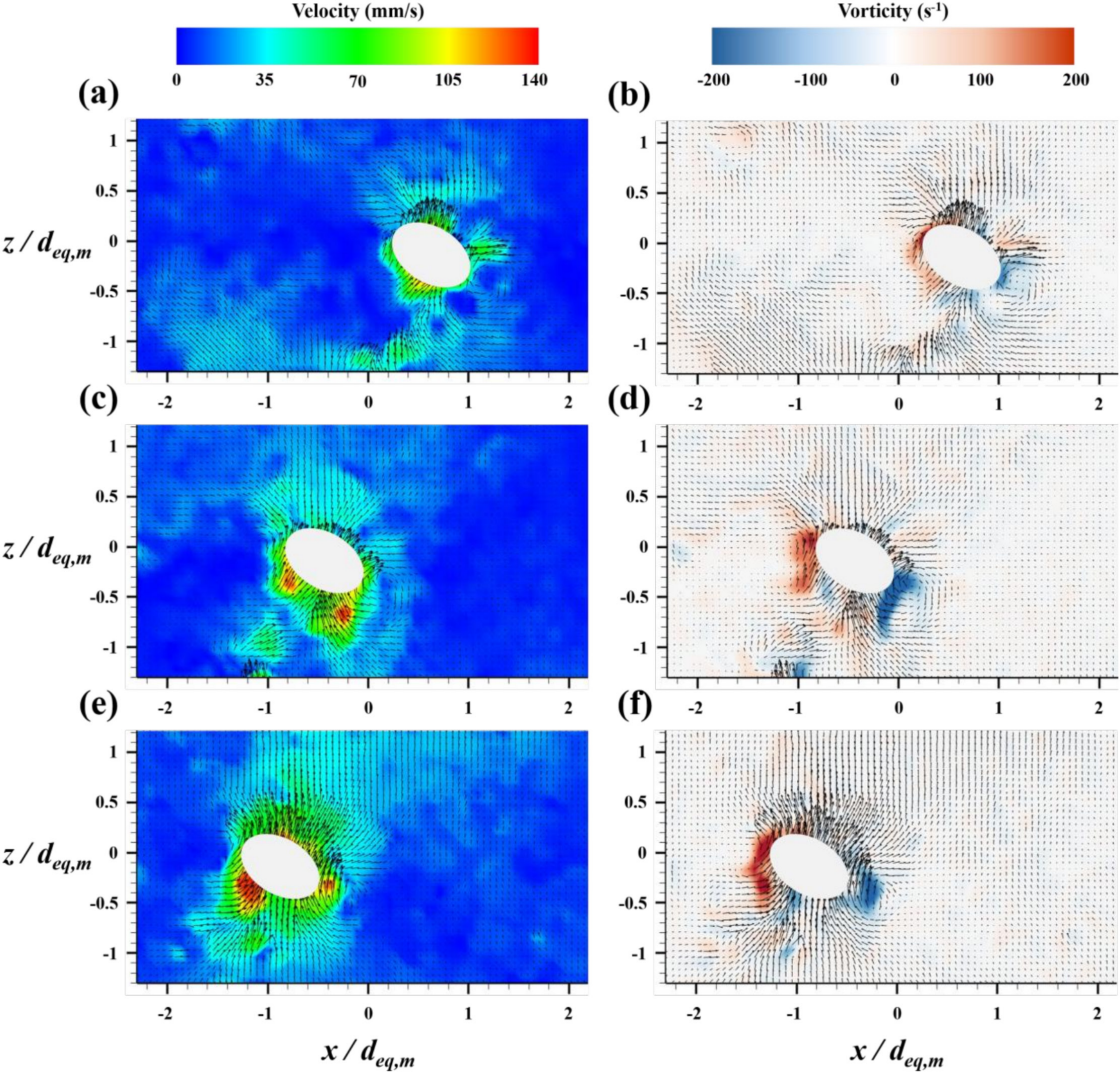
Velocity and vorticity fields induced by the bubble‐chain system under the condition of *d*_*eq,m*_ = 3.21 mm, evaluated when an individual bubble reaches the reference position *z*/*d*_*eq,m*_ = 0 within the FOV. Panels (a) and (d) show the velocity and vorticity distributions for *N* = 1, (b) and (e) for *N* = 5, and (c) and (f) for *N* = 10, respectively. For each *N*, the flow fields represent conditionally averaged results obtained by averaging three instantaneous velocity fields calculated from three consecutive PIV image pairs (corresponding to six raw images), centered on the moment when the bubble reaches *z*/*d*_*eq,m*_ = 0.

For *N* = 1, the flow field extended from *z*/*d*_*eq,m*_ = −0.5 to 0.5 in the vertical direction and from *x*/*d*_*eq,m*_ = 0 to 1 in the horizontal direction (Figure 7a). Under this condition, PS particles underwent initial displacement upon direct collision with the bubble, followed by re‐entrainment into the wake through surrounding vortex structures. This process established a continuous upward transport pathway along the *z*‐direction, consistent with the flotation mechanism reported by Choi and Park (2022) (Figures 6 and 7b).

For *N* = 5, the flow field expanded in both the horizontal and vertical directions. The vertical range extended from *z*/*d*_*eq,m*_ = −1 to 0.8, while the horizontal extent ranged from *x*/*d*_*eq,m*_ = −1 to 0.4 (Figure 7c). Compared with the single‐bubble case, this condition produced a broader and more energetic flow structure. The observed expansion corresponded to overlapping vortex structures generated by consecutively rising bubbles, as illustrated in Figure 7d.

For *N* = 10, the vertical extent further increased, spanning from *z*/*d*_*eq,m*_ = −1 to 1. Distinct spatial distributions emerged between the front and wake regions of the bubble chain. In the front region, the horizontal extent ranged from *x*/*d*_*eq,m*_ = −1 to 2, whereas in the wake region it was confined between *x*/*d*_*eq,m*_ = −1.8 to 0 (Figure 7e).

## 4 Discussion

### 4.1 Hydrodynamic Implications of Bubble Path Instability

The observed path instability extends the hydrodynamic influence of rising bubbles beyond a narrow vertical wake. As bubbles deviate laterally, momentum is redistributed over a broader region, promoting the formation of alternating vortical structures. This exhibits characteristics similar to the wake‐shear interaction mechanism reported by Tomiyama et al. (2002).

Consequently, particle entrainment can occur even without direct bubble–particle collision, indicating that nonlinear bubble trajectories, mediated by deformation‐induced transverse lift, contribute directly to enhanced hydrodynamic lifting. However, because the experiments were conducted in a finite laboratory‐scale tank, subtle wall‐induced modifications to bubble trajectories and wake development cannot be entirely excluded. The present interpretation of path instability should therefore be understood within the spatial scale of the experimental configuration.

### 4.2 Transition From Single‐Bubble Attachment to Flow‐Dominated Transport

Classical single‐bubble studies report that smaller bubbles enhance particle–bubble attachment efficiency (Hewitt et al. 1995; Ralston et al. 1999). In contrast, the present multibubble results show increased axial particle displacement with larger bubbles, indicating a shift from attachment‐controlled behavior to flow‐dominated transport. Similar macroscopic transport enhancement has been observed in two‐dimensional bubble‐chain systems (Yeo and Park 2024).

As successive bubbles generate overlapping vortical fields, momentum is redistributed over an extended region, promoting particle displacement beyond direct collision mechanisms. However, it should be noted that the present interpretation is based on two‐dimensional PIV measurements within a localized FOV; thus, fully three‐dimensional interactions and larger scale unsteady structures may not be completely resolved.

### 4.3 Influence of Surfactant Absence on Bubble Dynamics

No frother or surfactant was employed in the present experiments. In practical flotation systems, frothers are added to stabilize bubbles and suppress lateral oscillations, thereby modifying rise dynamics and wake structure (Tagawa et al. 2014; Takagi and Matsumoto 2011). Under the clean‐water conditions adopted here, bubbles retain highly mobile interfaces and exhibit enhanced path instability and vortex shedding. Consequently, the observed flow‐field expansion represents a hydrodynamically intensified scenario. In chemically conditioned flotation systems, these effects are expected to be moderated, although the fundamental bubble‐chain‐induced lifting mechanism remains physically relevant.

### 4.4 Scope and Applicability to Real DAF Systems

The present experiments were conducted under quiescent, clean‐water conditions without imposed bulk flow, turbulence, or chemical conditioning. In practical DAF systems, background turbulence, suspended solids, and coagulants may alter bubble trajectories, wake coherence, and attachment efficiency (De Somer et al. 2024; Mathai et al. 2020). Consequently, the absolute magnitude of particle displacement observed here may differ under process‐scale conditions.

Nevertheless, the bubble‐chain‐induced hydrodynamic lifting mechanism identified in this study remains physically relevant in realistic flotation environments, although its quantitative impact may be moderated under process‐scale conditions. Consequently, the absolute magnitude of particle displacement observed here should be interpreted within the present experimental configuration.

Furthermore, quantitative recovery or removal efficiencies were not evaluated, as the study was designed to isolate hydrodynamic transport mechanisms rather than process‐scale separation performance. Under millimeter‐scale bubble conditions, recovery cannot be uniquely defined without introducing additional system‐level constraints such as collection boundaries or residence‐time criteria, which were not imposed in the present configuration.

## 5 Conclusion

We experimentally elucidated the role of bubble‐chain‐induced flow fields in governing the transport of MPs using PIV. Successive bubble generation broadened the hydrodynamic domain in both horizontal and vertical directions, thereby entraining surrounding particles and promoting their upward motion. As the number of bubbles increased, vertical displacement and total travel distance rose by up to 44.8% and 48.7%, respectively.

These results indicate that collective bubble‐chain dynamics enhance particle transport through flow‐field amplification beyond isolated bubble–particle attachment mechanisms. The findings provide mechanistic insight into hydrodynamically driven lifting in millimeter‐scale bubble systems. From a practical perspective, this understanding may assist in interpreting transport behavior in DAF processes and in refining strategies for microplastic removal where bubble‐chain effects are present.

## Author Contributions

**Hyeok Jun Nam:** conceptualization, methodology, investigation, formal analysis, data curation, visualization, writing – original draft. **Sung Jun Han:** methodology, validation, investigation, data curation, formal analysis, writing – original draft. **Jeong Jae Kim:** conceptualization, supervision, project administration, resources, funding acquisition, writing – review and editing, correspondence.

## Conflicts of Interest

The authors declare no conflicts of interest.

## Supporting information


**Figure S1:** Scanning electron microscope (SEM) image of PS particles used in the experiment.
**Figure S2:** Volume‐based particle size distribution of PS particles obtained from laser diffraction analysis, presented as the percentage volume density versus particle diameter.
**Table S1:** Results of laser diffraction particle size analysis for polystyrene (PS) particles employed as seeding tracers in the experiment.
**Table S2:** wer70374‐sup‐0001‐Supporting_Information.docx. *d*_*eq*_ and *χ* of individual bubbles constituting the bubble‐chain system when using the needle with an inner diameter of 0.32 mm.
**Table S3:** wer70374‐sup‐0001‐Supporting_Information.docx. *d*_*eq*_ and *χ* of individual bubbles constituting the bubble‐chain system when using the needle with and inner diameter of 0.23 mm.

## Data Availability

The data that support the findings of this study are available from the corresponding author upon reasonable request.
