# Supplementary material for: Enhanced Microplastic Flotation: Unraveling the Role of Bubble‐Chain Hydrodynamics via PIV Analysis
Source: Water Environ Res. 2026 Apr 8;98(4):e70374. doi: 10.1002/wer.70374 (PMC13062615; doi:10.1002/wer.70374)
Supplement: Supplementary file 1 — Figure S1: Scanning electron microscope (SEM) image of PS particles used in the experiment. Figure S2: Volume‐based particle size distribution of PS particles obtained from laser diffraction analysis, presented as the percentage volume density versus particle diameter. Table S1: Results of laser diffraction particle size analysis for polystyrene (PS) particles employed as seeding tracers in the experiment. Table S2: wer70374‐sup‐0001‐Supporting_Information.docx. d eq and χ of individual bubbles constituting the bubble‐chain system when using the needle with an inner diameter of 0.32 mm. Table S3: wer70374‐sup‐0001‐Supporting_Information.docx. d eq and χ of individual bubbles constituting the bubble‐chain system when using the needle with and inner diameter of 0.23 mm. [file WER-98-e70374-s001.docx]

**Supplementary Information**

**Enhanced microplastic flotation: Unraveling the role of bubble-chain hydrodynamics via PIV Analysis**

Hyeok Jun Nam^a, †^, Sung Jun Han^b, †^, Jeong Jae Kim^a, *^

^a^Department of Mechanical Engineering, Hanbat National University, Daejeon, Korea 37673

^b^Division of Environmental Science and Engineering, Pohang University of Science and Technology, Pohang, Korea 37673

†Equally contributed authors

*Corresponding author

Jeong Jae Kim, Department of Mechanical Engineering, Hanbat National University, 125, Dongseodaero, Yuseong-Gu, Daejeon, Korea 34518.

e-mail: jjk11@hanbat.ac.kr

**Contents**

**S1. Supplementary Tables**

**Table S1** Results of laser diffraction particle size analysis for polystyrene (PS) particles employed as seeding tracers in the experiment

**Table S2** *d_eq_* and χ of individual bubbles constituting the bubble-chain system when using the needle with an inner diameter of 0.32 mm

**Table S3** *d_eq_* and *χ* of individual bubbles constituting the bubble-chain system when using the needle with and inner diameter of 0.23 mm

**S2. Supplementary Figures**

**Figure S1** Scanning electron microscope (SEM) image of PS particles used in the experiment

**Figure S2** Volume-based particle size distribution of PS particles obtained from laser diffraction analysis, presented as the percentage volume density versus particle diameter

**S1. Supplementary Tables**

**Table S1.** Results of laser diffraction particle size analysis for polystyrene (PS) particles employed as seeding tracers in the experiment

| **Concentration** | 0.0103 % |
| --- | --- |
| **Span** | 1.218 |
| **Uniformity** | 0.379 |
| **Specific Surface Area** | 442.4 m^2^/kg |
| **D [3,2]** | 13.6 μm |
| **D [4,3]** | 19.0 μm |
| **Dv (10)** | 8.93 μm |
| **Dv (50)** | 18.1 μm |
| **Dv (90)** | 31.0 μm |

**Table S2.** *d_eq_* and χ of individual bubbles constituting the bubble-chain system when using the needle with an inner diameter of 0.32 mm

| *N*th | 1 | 2 | 3 | 4 | 5 | 6 | 7 | 8 | 9 | 10 |
| --- | --- | --- | --- | --- | --- | --- | --- | --- | --- | --- |
| *d_eq_* (mm) | 2.86 | 3.06 | 3.13 | 3.29 | 3.29 | 3.23 | 3.46 | 3.09 | 3.49 | 3.21 |
| *χ* | 2.76 | 2.6 | 2.51 | 2.14 | 1.92 | 2.26 | 2 | 2.55 | 2.46 | 2.06 |

**Table S3.** *d_eq_* and *χ* of individual bubbles constituting the bubble-chain system when using the needle with an inner diameter of 0.23 mm

| *N*th | 1 | 2 | 3 | 4 | 5 | 6 | 7 | 8 | 9 | 10 |
| --- | --- | --- | --- | --- | --- | --- | --- | --- | --- | --- |
| *d_eq_* (mm) | 2.70 | 2.53 | 2.62 | 2.61 | 2.76 | 2.87 | 3.11 | 2.92 | 2.93 | 3.08 |
| *χ* | 2.66 | 2.22 | 3.08 | 2.16 | 3.08 | 2.33 | 2.39 | 2.82 | 2.70 | 2.34 |

**S2. Supplementary Figures**


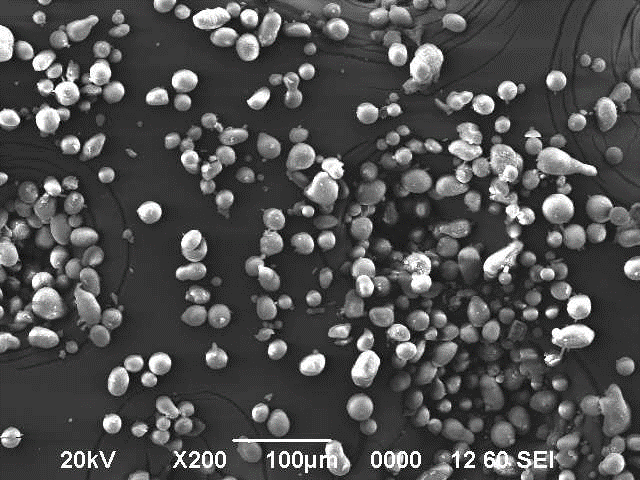


**Figure S1.** Scanning electron microscope (SEM) image of PS particles used in the experiment


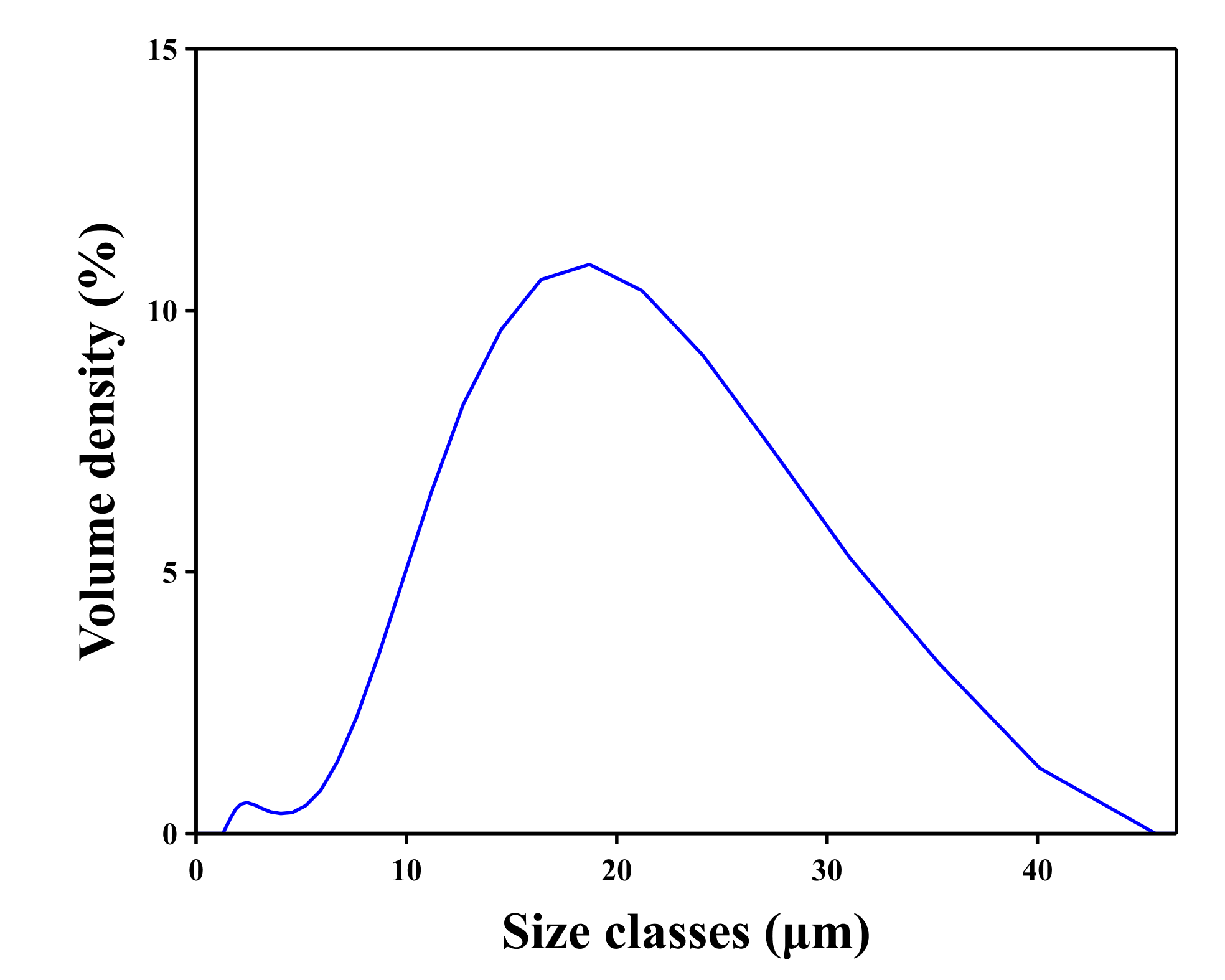


**Figure S2.** Volume-based particle size distribution of PS particles obtained from laser diffraction analysis, presented as the percentage volume density versus particle diameter
